# Primary Ewing′s Sarcoma of the Sinonasal Region: A Rare Clinical Encounter

**DOI:** 10.1155/crip/4920419

**Published:** 2026-03-06

**Authors:** Ujjwal Sangroula, Prajeeta Bhandari, Ratan Shah, Prajjwol Luitel, Sadmarg Thakur, Monica Shah, Manoj Tamang

**Affiliations:** ^1^ Department of Pathology, National Medical College and Teaching Hospital, Birgunj, Nepal, nmcbir.edu.np; ^2^ Department of Pathology, B.P. Koirala Memorial Cancer Hospital, Bharatpur, Nepal, bpkmch.org.np; ^3^ Maharajgunj Medical Campus, Tribhuvan University Teaching Hospital, Kathmandu, Nepal, teachinghospital.org.np; ^4^ Nepal Medical College and Teaching Hospital, Kathmandu, Nepal

**Keywords:** case report, Ewing′s sarcoma, sinonasal tract, small round cell tumor

## Abstract

Ewing′s sarcoma (EWS) is a highly aggressive tumor of neuroectodermal origin, rarely occurring in the sinonasal tract, particularly in adults. We report a 27‐year‐old male with long‐standing nasal obstruction, initially misdiagnosed as nasal chondromesenchymal hamartoma. Surgical excision revealed a destructive, vascular sinonasal mass. Histology showed sheets of small round cells within a sclerotic stroma, and immunohistochemistry positivity for CD99, vimentin, cyclin D1, BCL2, and NKX2.2 confirmed EWS. This case underscores the diagnostic difficulty of sinonasal small round cell tumors and the necessity of thorough histopathological and immunohistochemical analysis.

## 1. Introduction

Ewing′s sarcoma (EWS) is a highly malignant and rare small round cell tumor which belongs to the primitive neuroectodermal tumor (PNET) class [1]. It presents in both skeletal and extraskeletal forms, with the skeletal type being more common and typically involving the long bones of the extremities ranking second after osteosarcoma. It contributes to approximately 4%–6% of all primary bone malignancies [1, 2]. Extraskeletal EWS typically develops in soft tissue, most often affecting regions such as the lower extremities, paravertebral area, chest wall, and retroperitoneum. In contrast, its presence in the head and neck region is quite rare, representing only about 1%–4% of all reported cases [3, 4]. Cases originating in the sinonasal tract are particularly uncommon. EWS is characterized by a chromosomal translocation that involves the EWS gene located on chromosome 22q12. EWS is most commonly associated with a chromosomal translocation involving the EWS gene on chromosome 22q12 and the friend leukemia virus integration site 1 (FLI‐1) gene on chromosome 11q24. This results in the t(11;22)(q24;q12) fusion, which is identified in about 85% of EWS cases and in over 90% of its extraosseous variants. A comprehensive search across databases including the Cochrane Library, Web of Science, PubMed, SCOPUS, and CINAHL revealed that, as of November 2024, roughly 93 cases of EWS originating in the sinonasal region have been documented in the literature [5]. The average age at diagnosis is 26.4 years, with a slight male predominance [1, 5, 6]. Due to its rarity and resemblance to other small round cell tumors, accurate diagnosis is challenging and requires thorough evaluation.

Adhering to the CARE (Case Report) guidelines, we present the case of a 27‐year‐old man presented for nasal blockage for 3 years.

## 2. Case Presentation

A 27‐year‐old male presented with a 3‐year history of nasal blockage without other symptoms. Radiological evaluation revealed an aggressive sinonasal lesion. CT scan findings were suggestive of an aggressive sinonasal ossifying fibroma or a neoplastic lesion. MRI revealed an enhancing sinonasal mass centered in the sphenoid sinuses and the right posterior ethmoid air cells. The lesion extended into the right nasopharyngeal and parapharyngeal spaces, with evidence of infiltration into the clivus and sella. Based on these findings, further evaluation and histopathological examination (HPE) were recommended. An incisional biopsy was performed and sent for histopathology. Microscopic examination revealed well‐demarcated mature cartilage in a predominantly myxoid stroma with focal osteoclast‐like giant cells, scattered spindle cells, collagen fibers, and a few benign nasal glands. The initial provisional impression was given as nasal chondromesenchymal hamartoma (NCMH).

**Figure 2:**
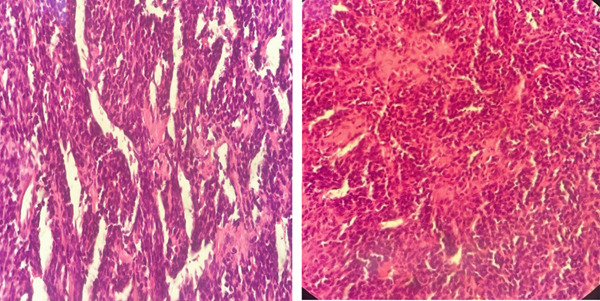
Photomicrograph of tumor cells with uniform, round nuclei, stippled chromatin, inconspicuous nucleoli, indistinct cell membranes, and scant eosinophilic cytoplasm (H and E, 400×).

Subsequently, the patient underwent wide local excision (WLE) of the right sinonasal mass via maxillary swing approach. Intraoperatively, the mass appeared vascular and proliferative, extending anteriorly into the nasal cavity, laterally into the pterygoid fossa, and posteriorly up to the clivus and cavernous sinus. The mass also involved the nasopharynx, torus tubarius, maxillary sinus, sphenoid sinus, and ethmoidal sinus. Microscopy demonstrated areas of hemorrhage and calcification (Figure 1). Sections also showed sheets of small, uniform round blue cells set in a sclerotic stroma with focal ossification (Figure 2). The tumor cells had indistinct borders, stippled chromatin, inconspicuous nucleoli, and scant eosinophilic cytoplasm, and they infiltrated adjacent tissues. Mitoses were rare and no necrosis was seen. There was no lymphovascular or perineural invasion.

Figure 1(a) Photomicrograph of tumor mass with normal adjacent nasal mucosal glands (H and E, 40×). (b) Photomicrograph of tumor cells with infiltration and destruction of bone (H and E, 40×).

**Figure:**
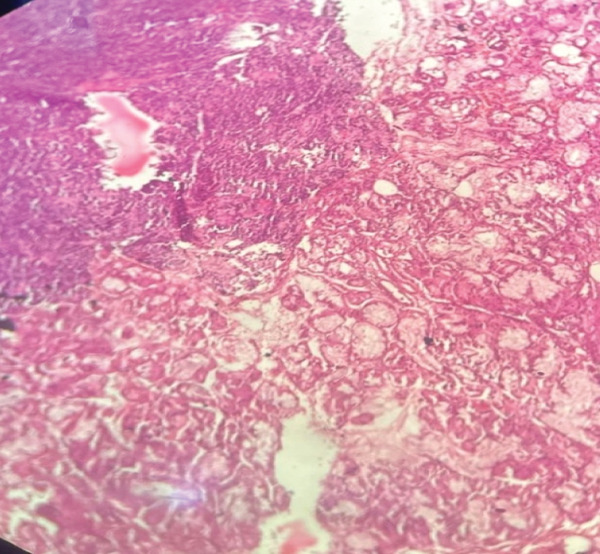
(a)

**Figure:**
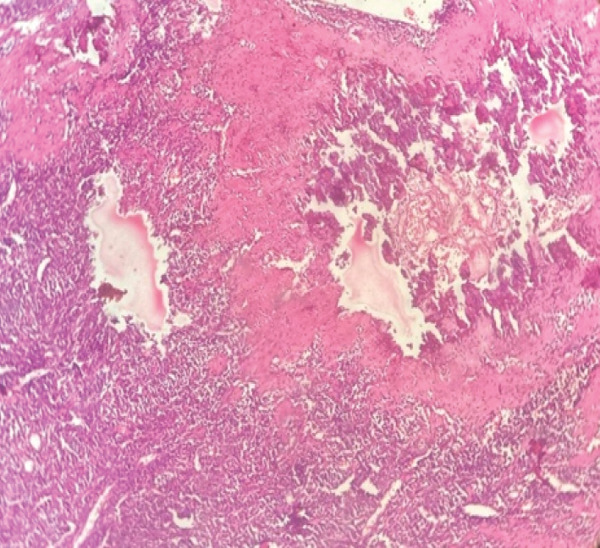
(b)

The histomorphological features were consistent with a malignant small round cell tumor of probable neuroendocrine origin. The differential diagnosis included olfactory neuroblastoma, small‐cell neuroendocrine carcinoma, embryonal rhabdomyosarcoma, and extranodal NK/T cell lymphoma. Immunohistochemistry (IHC) was advised for confirmation.

IHC revealed tumor cells positive for vimentin, cluster of differentiation (CD99), cyclin D1, B cell lymphoma (BCL2), and NKX2.2, whereas negative for cytokeratin (CK), synaptophysin, CD45, Wilms tumor (WT1), desmin, transducin‐like enhancer of split (TLE1), BCL6 corepressor (BCOR), Special AT‐rich sequence‐binding protein (SATB2), ETS‐related gene (ERG), p63, S100, and CD34. The Ki‐67 proliferation index was up to 20%. Based on the histomorphological features and IHC profile, a final diagnosis of EWS of sinonasal tract was made.

One month following surgery, the patient presented with a recurrent/residual lesion locally, which was confirmed by contrast‐enhanced CT (CECT). The patient was treated with four doses of chemotherapy (VDC‐IE: Vincristine, doxorubicin, and cyclophosphamide, alternating with ifosfamide and etoposide), as repeat surgery was not feasible. Sequentially, radiotherapy was also done after thorough discussion with the oncologist although there was significant risk for damage to optic structures and possibly the brainstem.

## 3. Discussion

Ewing′s sarcoma (EWS) was originally identified by James Ewing in 1921, who described it as a “diffuse endothelioma of bone” [7]. In 2022, WHO Classification of Head and Neck Tumors introduced a new approach by placing all soft tissue tumors, including Ewing sarcoma, into a separate, dedicated chapter rather than categorizing them by anatomical site. EWS is now listed under “undifferentiated small round cell sarcomas of bone and soft tissue,” acknowledging that approximately 10% of cases—whether arising in bone or soft tissue—can present in the head and neck region, including the sinonasal tract [8]. Sinonasal Ewing sarcoma (SNES) poses a clinical diagnostic challenge because it typically manifests with vague symptoms like nasal blockage, epistaxis, and facial swelling. As a result, diagnosis is often delayed, emphasizing the importance of maintaining a high index of suspicion and including SNES in the differential diagnosis when nasal symptoms persist despite routine treatment [4, 6, 9]. Additional signs and symptoms often arise from the tumor′s mass effect. Notably, when the lesion extends into the periorbital region, patients may experience proptosis, swelling around the eyes, and reduced visual acuity. Furthermore, metastatic disease is present in approximately 15%–30% of cases at the time of initial diagnosis [2].

Within the head and neck region, EWS most frequently affects the maxillary sinus, mandible, and skull base. Less commonly, it can also involve areas like the orbital roof and nasal cavities [5, 10]. EWS affecting both the sphenoid sinus and ethmoid air cells is extremely uncommon, as seen in this case, with only a handful of similar cases documented in the existing literature [10, 11]. Few cases are found in literature that originating from the sphenoid sinus and involving the sphenoid bone [6]. Similarly, primary tumors of the ethmoid sinus, particularly the posterior ethmoid air cells, have been documented in only a small number of case reports, often extending into adjacent areas like the orbit or sphenoid sinus [10, 11]. Cases arising simultaneously from both the sphenoid sinus and posterior ethmoid air cells are extremely uncommon and are typically described as part of a larger sinonasal or skull base involvement rather than isolated origins. In most of these cases, the tumor had locally extended into adjacent areas, including the maxillary and frontal sinuses, nasal cavity, nasal septum, orbit, and skull base. Ethmoidal sinus tumors appeared more likely to invade the orbit and cranium due to the small size and thin bony walls (lamina papyracea and skull base) of the ethmoid sinus, allowing easier spread. In contrast, the maxillary sinus, with its larger space and thicker bony walls, shows less locally aggressive spread. When intracranial extension is present, the middle cranial fossa is most commonly involved, as in our case [6, 11–13].

Molecularly defined EWS displays a range of histologic appearances, but about 80% shows classical features of diffuse sheets of small, uniform round cells with fine chromatin, inconspicuous nucleoli, and scant amphophilic or clear cytoplasm. Necrosis and mitoses are common [6, 10, 14, 15]. Nonclassic variants include the PNET pattern with Homer Wright rosette (often confused with neuroblastoma) [16], occasional bone formation (mimicking osteosarcoma), and clear cytoplasm due to glycogen. Other rare patterns include alveolar (resembling rhabdomyosarcoma), large cell (with eosinophilic cytoplasm and mild pleomorphism), spindle cell, nested epithelioid (mimicking neuroendocrine tumors in adults) [6, 15], and adamantinoma‐like variant showing squamoid features, often in head and neck [17]. A distinguishing histochemical feature is the presence of intracytoplasmic glycogen, highlighted by periodic acid‐Schiff (PAS)–positive and PAS‐D negative staining [2, 16].

The histopathological differential diagnosis of mass in the sinonasal tract includes a wide array of small round cell tumors like olfactory neuroblastoma, undifferentiated carcinoma, embryonal rhabdomyosarcoma, sinonasal melanoma, mesenchymal chondrosarcoma, small‐cell osteosarcoma, lymphoma, acute leukemia, and small‐cell neuroendocrine carcinoma, which can be challenging for diagnosis as they overlap histopathological morphology.

In IHC, EWS commonly shows strong, diffuse membranous positivity for CD99 [1, 6, 10, 15, 16], which is nonspecific but a characteristic finding. NKX2.2 and PAX7 are more specific immunohistochemical markers that are expressed in approximately 90%–99% of EWS cases and are associated with the presence of Ewing sarcoma breakpoint region (EWSR) 1 fusion genes. CD99, NKX2.2, and PAX7 together form a highly specific diagnostic panel [14, 15, 18–20].

NKX2.2 may show reduced sensitivity in large or decalcified specimens and can also be expressed in tumors like olfactory neuroblastoma or small‐cell carcinoma. PAX7 may overlap with rhabdomyosarcoma and BCOR‐altered sarcomas. These markers remain useful across EWS subtypes and fusion types. Even with molecular testing, IHC is essential especially in equivocal Fluorescence In situ hybridization (FISH) cases, such as EWSR1::ERG fusions, where ERG staining may assist [15, 20].

About 30% of EWS express keratin, which can mimic neuroendocrine tumors. Ewing cells also express FLI‐1, CD56, CD45, S‐100 protein, synaptophysin, vimentin, and desmin [20, 21] whereas chromogranin A is rarely expressed. BCL2 expression can be observed in sinonasal EWS; however, this finding is not specific, as BCL2 is also commonly expressed in other small round cell tumors, including lymphomas, synovial sarcoma, desmoplastic small round cell tumors, and rhabdomyosarcoma [22]. Markers like S100, desmin, myogenin, and MyoD1 are usually negative, which helps to exclude tumors that mimic the morphology. c‐KIT, cyclin D1, and claudin‐1 may be positive but lack diagnostic specificity [15, 21].

Histopathology and IHC are typically sufficient for diagnosing extraskeletal Ewing sarcoma (EES). However, molecular genetic testing, such as reverse transcriptase polymerase chain reaction (RT‐PCR) or FISH, becomes crucial in atypical or ambiguous cases. The two most frequent translocations associated with EES and the Ewing sarcoma family of tumors (ESFT) are t(11;22)(q24;q12) and t(21;22)(q22;q12). The t(11;22) translocation, found in about 90% of cases, results in an EWSR1‐FLI1 fusion, producing a chimeric transcription factor with the DNA‐binding domain of FLI1. The t(21;22) translocation leads to an EWSR1–ERG fusion and also generates an oncogenic transcription factor that promotes tumor survival by inhibiting apoptosis. Other rare translocations involving the EWSR1 gene on chromosome 22 have also been reported [2, 6, 15, 18, 23–25].

Surgical removal of the tumor, along with radiotherapy and chemotherapy, is regarded as an effective treatment strategy for EWS and can result in a cure rate of ≥ 50% [25]. However, due to the complex anatomy of the sphenoid bone and sphenoid sinus, achieving adequate surgical margins during tumor excision can be challenging as in our case. Therefore, radiotherapy is often used postoperatively to enhance local disease control and may also serve as an alternative to surgery in patients who are not suitable candidates for surgical intervention [25].

In our case, we could not follow up the patient even after 1 year.

## 4. Conclusion

SNES is a rare and aggressive tumor that poses diagnostic and therapeutic challenges due to its nonspecific symptoms and resemblance to other small round cell neoplasms. Making an accurate diagnosis can be difficult, as many small round cell tumors share similar features. It requires careful evaluation through histopathology and IHC, supported by molecular testing. The presence of the EWSR1‐FLI1 gene fusion serves as a crucial diagnostic marker for confirming EWS. Complete surgical excision is often limited by the complex anatomy of the sinonasal region. Therefore, multimodal approach involving surgery, chemotherapy, and radiotherapy remains the cornerstone of treatment, although robust comparative data from large case series is lacking due to the rarity of reported cases in the literature. Early diagnosis and tailored treatment can significantly improve outcomes and preserve quality of life.

## Author Contributions

Conceptualization: Ujjwal Sangroula, Prajeeta Bhandari, Ratan Shah, Prajjwol Luitel, Sadmarg Thakur, Monica Shah, and Manoj Tamang; patient management: Ujjwal Sangroula, Prajeeta Bhandari, and Ratan Shah; writing—original draft: Ujjwal Sangroula, Prajeeta Bhandari, Ratan Shah, Prajjwol Luitel, Sadmarg Thakur, Monica Shah, and Manoj Tamang; writing—review and editing: Ujjwal Sangroula, Prajeeta Bhandari, Ratan Shah, Prajjwol Luitel, Sadmarg Thakur, Monica Shah, and Manoj Tamang; visualization and supervision: Ujjwal Sangroula, Prajeeta Bhandari, Ratan Shah, Prajjwol Luitel, Sadmarg Thakur, Monica Shah, and Manoj Tamang.

## Funding

No funding was received for this manuscript.

## Disclosure

We assure that the authors are ultimately responsible for and accountable for the contents of the work.

## Ethics Statement

The study was exempt from ethical approval.

## Consent

Written informed consent was obtained from the patient′s caregiver for publication and any accompanying images. A copy of the written consent is available for review by the editor in chief of this journal on request.

## Conflicts of Interest

The authors declare no conflicts of interest.

## Data Availability

The data that support the findings of this study are available on request from the corresponding author. The data are not publicly available due to privacy or ethical restrictions.
